# Supplementary material for: Multi‐dataset identification of innovative feature genes and molecular mechanisms in keratoconus
Source: J Cell Mol Med. 2024 Sep 19;28(18):e70079. doi: 10.1111/jcmm.70079 (PMC11412914; doi:10.1111/jcmm.70079)
Supplement: Supplementary file 2 — Material S2. [file JCMM-28-e70079-s002.zip › 7.GSEA analysis/1.ARL11/KEGG_GLYCOSAMINOGLYCAN_BIOSYNTHESIS_KERATAN_SULFATE.html]

Details for gene set KEGG\_GLYCOSAMINOGLYCAN\_BIOSYNTHESIS\_KERATAN\_SULFATE[GSEA]

|  || Dataset | ARL11.group.cls#h\_versus\_l |
| Phenotype | group.cls#h\_versus\_l |
| Upregulated in class | l |
| GeneSet | KEGG\_GLYCOSAMINOGLYCAN\_BIOSYNTHESIS\_KERATAN\_SULFATE |
| Enrichment Score (ES) | -0.33276188 |
| Normalized Enrichment Score (NES) | -0.954884 |
| Nominal p-value | 0.523166 |
| FDR q-value | 1.0 |
| FWER p-Value | 1.0 |
Table: GSEA Results Summary

  

Fig 1: Enrichment plot: KEGG\_GLYCOSAMINOGLYCAN\_BIOSYNTHESIS\_KERATAN\_SULFATE      
 Profile of the Running ES Score & Positions of GeneSet Members on the Rank Ordered List

  

| SYMBOL | TITLE | RANK IN GENE LIST | RANK METRIC SCORE | RUNNING ES | CORE ENRICHMENT || 1 | CHST2 | na | 2567 | 0.261 | 0.0639 | No |
| 2 | ST3GAL2 | na | 6403 | 0.126 | 0.0201 | No |
| 3 | B4GAT1 | na | 8463 | 0.084 | 0.0049 | No |
| 4 | B4GALT2 | na | 8950 | 0.076 | 0.0308 | No |
| 5 | CHST6 | na | 9306 | 0.070 | 0.0577 | No |
| 6 | ST3GAL1 | na | 11491 | 0.042 | 0.0167 | No |
| 7 | B4GALT1 | na | 21286 | -0.033 | -0.2478 | No |
| 8 | B3GNT7 | na | 24222 | -0.065 | -0.2978 | Yes |
| 9 | B4GALT3 | na | 25437 | -0.082 | -0.2895 | Yes |
| 10 | B4GALT4 | na | 25675 | -0.085 | -0.2513 | Yes |
| 11 | CHST4 | na | 27866 | -0.127 | -0.2471 | Yes |
| 12 | FUT8 | na | 27972 | -0.130 | -0.1814 | Yes |
| 13 | B3GNT2 | na | 29568 | -0.174 | -0.1353 | Yes |
| 14 | ST3GAL3 | na | 31188 | -0.227 | -0.0619 | Yes |
| 15 | CHST1 | na | 32838 | -0.310 | 0.0544 | Yes |
Table: GSEA details [plain text format]

  

Fig 2: KEGG\_GLYCOSAMINOGLYCAN\_BIOSYNTHESIS\_KERATAN\_SULFATE      
 Blue-Pink O' Gram in the Space of the Analyzed GeneSet

  

Fig 3: KEGG\_GLYCOSAMINOGLYCAN\_BIOSYNTHESIS\_KERATAN\_SULFATE: Random ES distribution      
 Gene set null distribution of ES for **KEGG\_GLYCOSAMINOGLYCAN\_BIOSYNTHESIS\_KERATAN\_SULFATE**
